# Effects of Tissue Flossing on Athletic Performance Measures: A Systematic Review

**DOI:** 10.3390/sports12110312

**Published:** 2024-11-18

**Authors:** Scott W. Cheatham, Justin Nadeau, William Jackson, Russell Baker

**Affiliations:** 1 Department of Kinesiology, California State University Dominguez Hills, Carson, CA 90747, USA; 2 Department of Movement Sciences, University of Idaho, Moscow, ID 83844, USA; 3 Athletics Department, Colorado Christian University, Lakewood, CO 80226, USA; 4 Body Shop by Work Time Athletic Performance, Colorado Springs, CO 80920, USA; 5 WWAMI Medical Education Program, University of Idaho, Moscow, ID 83844, USA; 6 Idaho Office of Underserved and Rural Medical Research, University of Idaho, Moscow, ID 83844, USA

**Keywords:** massage, muscle, myofascial, treatment, band

## Abstract

The primary aim of this study was to investigate the efficacy of tissue flossing on athletic performance measures. A secondary aim was to explore the efficacy of tissue flossing when applied to a joint or soft tissue (i.e., muscle belly) on athletic performance measures. An article search was completed in the PubMed/MEDLINE, EBSCO, SCOPUS, and OneSearch electronic databases up to May 2024. Studies were included if they used tissue flossing as a primary intervention among healthy participants and used one or more athletic performance measures as an outcome. Exclusion criteria included studies that did not investigate tissue flossing on athletic performance measures among healthy participants, studies that used tissue flossing for blood flow restriction training, case studies, narrative reviews, dissertations, conference proceedings, and papers written in a language other than English. Eighteen articles and 559 total participants were included in the final analysis. Study quality was assessed by two independent reviewers using the Downs and Black Checklist and the Oxford Centre for Evidence-based Medicine. The major findings suggest that a single tissue flossing treatment ranging from 2 to 10 min that includes active single joint or active closed chain exercises may enhance post-intervention muscle strength, jump performance, and balance up to 45 to 60 min post-intervention. Tissue flossing to a joint or soft tissue both produced mixed results among studies, a definitive answer on which technique is superior cannot be determined at this time. Further direct comparison studies are needed for these two techniques.

## 1. Introduction

**Figure 1:**
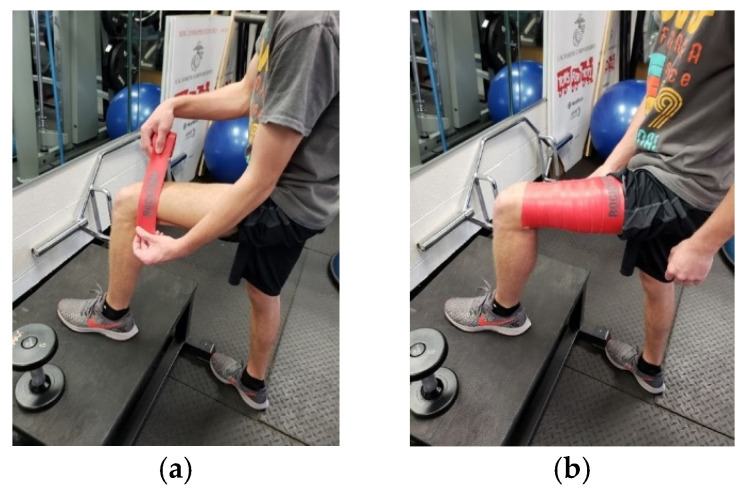
(**a**) Application of tissue flossing wrapping technique for the thigh-*first step*. (**b**) Application of tissue flossing wrapping technique for the thigh-*final step*.

Tissue flossing is a myofascial intervention used by healthcare professionals to reduce pain, increase myofascial mobility, improve joint ROM, and enhance athletic performance [1]. Introduced in 2013 by Starrett and Cordoza [2], tissue flossing consists of wrapping a latex tissue flossing band around a body region (joint or soft tissue) using a 50% overlapping circumferential pattern (distal to proximal), with a stretch ranging from 50% to 90% of the band maximal length (Figure 1) [1,2,3].

After applying the band, the patient performs up to a ten-minute treatment session that may include different active and passive movements of the wrapped body region [1].

Tissue flossing bands are commonly made of flat latex rubber (1.5 mm thick) and available in 2-inch (5.08 cm) and 4-inch (10.16 cm) widths with an average length of 7 feet long (213.36 cm) [1,3]. These bands have different architectural properties, such as width and thickness, than other flat latex resistance bands commonly used for fitness, rehabilitation, and sports performance [3,4,5]. Several companies manufacture tissue flossing bands with some manufacturers offering professional education on the technique [1].

The popularity of the intervention has stimulated more research, which has yielded an emerging body of evidence over the past 10 years. To date, two systematic reviews have been published that appraised studies regarding the effects of tissue flossing on sports and injury prevention, rehabilitation, and ankle dorsiflexion [6,7]. In 2021, researchers published a scoping review of tissue flossing that examined the effects on a healthy and impaired musculoskeletal system and included tissue flossing studies related to range of motion, performance parameters (e.g., strength or jump performance), pain and injury, disease, and recovery (e.g., delayed-onset muscle soreness) [8]. Since 2021, no other systematic or scoping reviews have examined the effects of tissue flossing on athletic performance measures such as muscle strength, jump performance, sprint performance, balance, and agility.

An evidence-based update regarding the efficacy of tissue flossing on athletic performance measures may reveal more insights into the utility of tissue flossing on these outcomes and promote continued research on this topic. Another point of interest is the specific post-intervention effects of tissue flossing techniques on the joint or soft tissue on athletic performance measures since healthcare professionals and researchers wrap both the joint and soft tissue [1,2,3]. The prior systematic and scoping reviews combined both techniques in their analysis and did not compare their individual post-intervention effects [6,7,8]. There is a need to appraise the current body of evidence and the effects of these two tissue flossing techniques on athletic performance measures. The primary aim of this systematic review was to investigate the efficacy of tissue flossing on athletic performance measures. A secondary aim was to explore the efficacy of tissue flossing when applied to a joint or soft tissue (i.e., muscle belly) on athletic performance measures.

## 2. Materials and Methods

**Figure 2:**
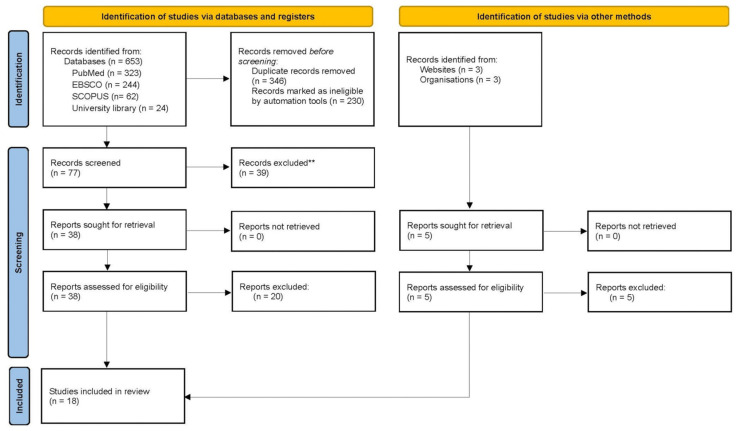
PRISMA flow diagram.

This systematic review was conducted according to the Preferred Reporting Items for Systematic Reviews and Meta-analyses (PRISMA) guidelines for reporting systematic reviews (Figure 2) [9]. This investigation was registered in the Prospero database (CRD42024511097).

### 2.1. Literature Search

The following online databases were searched up to May 2024: PubMed/MEDLINE, EBSCO, SCOPUS, and OneSearch. Direct searches of known websites and journals were also conducted to identify potential publications. The leading search terms were “Tissue flossing OR floss bands OR floss NOT dental NOT animal”. Other search terms (e.g., athletic, agility, balance, counterforce, dynamic, endurance, exercise, hop, height, jump, muscle, performance, power, sprint, speed, strength, static, quickness, and voodoo) were also used to identify additional studies. These search terms were adapted from a prior scoping review on tissue flossing [8].

### 2.2. Search Strategy

Two researchers (JN and JJ) conducted the same independent, blinded search of study titles and abstracts to ensure the accuracy and repeatability of the results. Upon completion, both individuals (JN and JJ) met to determine a final list of included studies. A third researcher (SWC) was available to resolve any disagreements. All three researchers (JJ, JN, and SWC) then independently completed a full-text review of qualifying studies and met to reach a consensus for inclusion. A fourth researcher (RB) was consulted to confirm inclusion if there was an impasse between authors. All authors agreed that the selected studies met the inclusion criteria. These study methods were adapted from a prior systematic review conducted by the authors [10].

### 2.3. Eligibility Criteria and Study Selection 

Studies considered for inclusion met the following criteria: (1) peer-reviewed, English language publications, (2) investigations that documented tissue flossing as a primary intervention for the upper extremity and/or lower extremity joints or soft tissues among healthy participants, and (3) tissue flossing investigations that used one or more athletic performance measures as an outcome such as muscular strength, jump performance, sprint performance, balance, and agility. Measures of strength were considered as follows: isokinetic testing (maximum voluntary isometric or eccentric contraction (MVIC/MVEC)), rate of force development (RFD), isokinetic peak torque (IPT), total work (TW), percentage of the repetitions maximum, rate of perceived exertion, power, and any known muscle performance tests. Measures of jump performance (e.g., double or single leg) were considered as follows: squat vertical jump, drop vertical jump, countermovement vertical jump (CMJ), single or double-leg hop, standing long jump or broad jump, or any known jump performance test. Measures of sprint performance were considered linear running tests with a specific distance (e.g., 5 m to 30 m). Measures of balance included the following: (1) static balance—single-leg balance test or any known test, (2) dynamic balance—single-leg squat, Y-balance Test, Star Excursion Balance Test, or any known test. Measures of agility included any known field test measuring agility performance. Studies that measured multiple outcomes were considered eligible but only the athletic performance measures were evaluated and reported due to the focus of this systematic review.

Exclusion criteria included studies that did not investigate tissue flossing on athletic performance measures among healthy participants, studies that used tissue flossing for blood flow restriction training, case studies, narrative reviews, dissertations, conference proceedings, and papers written in a language other than English.

### 2.4. Data Extraction 

A data extraction sheet was developed using Microsoft Word (Microsoft Corporation, Redmond, WA, USA), and extraction was performed by the investigators (JJ and JN). All the articles underwent a second assessment of data extraction by the investigator (SWC) to confirm agreement with the results. Extracted data included study characteristics (author(s), publication year, type of study), participant information (sample size, gender, age, activity level), intervention type and parameters, athletic performance measures, comparison groups, and study results.

### 2.5. Assessment of Study Quality 

The Downs and Black Checklist was used to assess the methodological quality of the selected studies. The checklist includes a total of 27 questions using 5 categories: reporting, external validity, internal validity (bias), internal validity (confounding) (selection bias), and power [11]. The scoring of question 27 (power) was modified based on prior systematic reviews from a possible score of 0 to 5 in the original checklist to a score of 0 or 1, depending on whether a power or sample size calculation was reported [12,13]. The checklist was scored out of 28 total points [12,13]. The ranges of corresponding scores were given a rating of excellent for scores between 26 and 28, good (20–25), fair (15–19), and poor (≤14) [10,14]. The selected studies were also analyzed using the Oxford Centre for Evidence-based Medicine (OCEBM) 2011 Levels of Evidence, a hierarchy of evidence scale. Each study was assigned a level of evidence in accordance with the OCEBM criteria [15].

### 2.6. Data Synthesis and Analysis 

Data were extracted, and a narrative analysis was performed. These data were pooled and analyzed based on the athletic performance measure, which included muscle strength, jump performance, sprint performance, balance, and agility. Qualifying data under each athletic performance measure were further categorized by the tissue flossing intervention technique of wrapping the joint or wrapping the soft tissues. A meta-analysis was not conducted. 

## 3. Results

The search initially identified 1306 citations from the electronic databases. A total of 346 duplicate articles were removed and 230 articles were ineligible. In total, 77 articles were screened, and 38 articles and 5 reports were retrieved and assessed for eligibility. A total of 20 articles and 5 reports were excluded due to not meeting inclusion criteria. A total of 18 studies were included in the final analysis (Figure 2). The reviewers’ Kappa value for the final 18 articles was 1.0 (perfect agreement).

### 3.1. Quality Assessment 

For the Downs and Black Checklist, seventeen studies scored in the good (20–25) category and one study scored in the excellent (26–28) category [10,14]. For the OCEBM criteria, six studies qualified as level II, and twelve studies qualified as level III (Table 1) [15].

### 3.2. Study Characteristics

All qualifying manuscripts yielded a total of 559 healthy adult participants (mean age of 22.50 years) with no medical issues that would exclude them from testing (Table 2). The following sections provide a narrative summary of the systematic analysis of accepted studies. Each section represents the specific athletic performance measure and the related studies categorized by tissue flossing intervention technique to the joint or soft tissue. Some studies are presented in both categories because the researchers investigated both techniques. *If available*, statistical significance levels were reported for each intervention outcome, along with the effect size. The effect size was interpreted using Cohen’s d, which uses the following scale: small (*d* = 0.2), moderate (*d* = 0.5), and large (*d* = 0.8) [34]. 

### 3.3. Muscle Strength

Nine studies investigated the post-intervention effects of a tissue flossing intervention on measures of muscle strength for the shoulder, knee, ankle, thigh, and calf [17,18,19,20,21,22,23,24,25] (Table 2). 

#### 3.3.1. Tissue Flossing: Joint

Three studies reported using a single-session tissue flossing intervention on the following joints: shoulder [16], knee [17], and ankle [20]. Two groups of researchers measured and monitored band stretch pressure (e.g., ≥150 mmHg) on the ankle joint [20] and shoulder joint [16] using a sub-band digital pressure sensor (e.g., Kikuhime™ pressure monitor, TT MediTrade, Sorø, Denmark). Another group applied the tissue flossing band using a hand-held dynamometer to measure stretch force to 40 N but did not report any monitoring of sub-band pressure [17].

All studies used isokinetic dynamometry to measure different aspects of strength, including MVIC [20], isokinetic peak torque (IPT) [16,20], and total work (TW) [16]. One study used the seated one-arm shot put test (OASPT) [16]. The total tissue flossing intervention time among studies ranged from 3 to 10 min [16,17,20]. All studies used a comparison group (Table 2).

Regarding results, two studies reported significant pre- to post-intervention changes after tissue flossing, and one reported non-significant post-intervention changes. For the *knee joint*, Chang et al. [17] reported immediate post-intervention changes with IPT for the quadriceps (*p* = 0.007, *d* = 1.94) and at a 20-min follow-up (*p* ≤ 0.001, *d* = 1.80) among active adult men. The researchers also compared tissue flossing to an elastic bandage condition. Tissue flossing produced greater post-intervention improvements in hamstring muscle force output (*p* = 0.022, *d* = 0.65) at the 20-min follow-up. For the *shoulder joint*, Angelopoulos et al. [16] reported immediate post-intervention changes in shoulder IPT (internal/external rotation @ 120°/210°/300°/s) (*p* value range of ≤ 0.001 to 0.004) and at a 45-min follow-up (*p* value range of ≤ 0.001 to 0.02) among amateur overhead adult athletes. The same researchers also reported immediate post-intervention changes in external rotation TW (@ 120°/210°/300°/s) immediately (*p*-value range ≤ 0.001 to 0.004) and at a 45-min follow-up (*p* ≤ 0.001 to 0.02) among athletes [16]. Shoulder internal rotation TW was non-significant (*p* = 0.261). Significant changes in the OASPT were also reported for both time points (*p* ≤ 0.005). The researchers also compared tissue flossing to instrument-assisted soft-tissue mobilization (IASTM), kinesiology tape (KT), and combined IASTM and tissue flossing. The researchers found that all interventions produced similar significant post-intervention changes immediately and at a 45-min follow-up when compared to the control group. For the *ankle joint*, Kalc et al. [20] reported immediate non-significant post-intervention changes after a tissue flossing intervention (mean pressure 293.8 ± 54.7 mmHg) for MVIC ankle plantarflexion (*p* = 0.203) and no changes at a 10 min follow-up (*p* = 0.568) among young martial arts athletes (e.g., ~16 years of age). The researchers did report a post-intervention reduction in soleus H reflex at both time points (Table 3) [20].

#### 3.3.2. Tissue Flossing: Soft Tissue

Seven studies reported the post-intervention effects of a single-session tissue flossing intervention that wrapped the soft tissue of the thigh (quadriceps and hamstrings) [18,19,22,23] or calf (plantar flexors) [20,21,25]. Six studies used and monitored band stretch pressure ranging between 100 mmHg and 210 mmHg, which was directly measured and monitored by a sub-band digital pressure sensor (e.g., Kikuhime™ pressure monitor, TT MediTrade, Sorø, Denmark; AMI-3037-SB-SET, AMI Techno., Ltd., Tokyo, Japan) [18,20,21,22] or an adapted sphygmomanometer [23,25]. One study only used the recommended stretch length (e.g., 50% band maximal length) and did not document any methods for measuring or monitoring band stretch pressure [19].

Seven studies used isokinetic dynamometry to measure different aspects of strength including MVIC or MVEC [20,21,23], RFD [21], power [19,25], TW [19], and IPT [19,25]. The total tissue flossing intervention time among studies ranged from 2 to 8 min [18,19,20,21,22,23,25]. All studies used a comparison group (Table 2).

Regarding results, four studies reported significant post-intervention changes after tissue flossing to the thigh and calf, and three studies reported non-significant post-intervention changes. For the *thigh region*, Konrad et al. [23] documented immediate post-intervention changes after a tissue flossing intervention (mean pressure 154.3 ± 13.3 mmHg) for MVIC of the knee extensors (*p* = 0.01, *d* = 0.77) when compared to the non-treatment control group among adult men. Vogrin et al. [18] examined the immediate and 30 min post-intervention changes after a tissue flossing intervention with knee extensor MVIC at three different band stretch pressures: control (20 mmHg), low (100 to 140 mmHg), and high (150 to 210 mmHg) among recreationally trained adults. The researchers documented that the low stretch pressure condition produced greater post-intervention changes in MVIC of the knee extensors immediately (*p* = 0.001) and at the 30-min follow-up (*p* = 0.01) when compared to the control (*d* = 0.78, 0.70) and high band stretch pressure (*d* = 0.80, 0.66) conditions. A study by Kaneda et al. [22] documented similar immediate post-intervention changes in MVEC of the knee extensors (*p* = 0.03, *d* = 0.43) and flexors (*p* = 0.02, *d* = 0.56) from a tissue flossing intervention (mean pressure 134 ± 10.2 mmHg) among adult men. The researchers also found greater post-intervention changes compared to the dynamic stretching group (MVEC extension *d* = 0.59. flexion *d* = 0.26) and active range of motion (AROM) control group (MVEC extension *d* = 0.43; MVEC flexion *d* = 0.56) [22]. Hamadus et al. [19] investigated the combination of tissue flossing with warm-up exercises. The researchers found immediate non-significant post-intervention changes in IPT, TW, and power (knee extension and flexion 60°/120°/180° s) (*p* > 0.05) when compared to a non-treatment control group among recreational adult athletes. 

For the *calf region*, Kaneda et al. [21] documented immediate significant post-intervention changes in RFD (plantar flexion @ 50/100/150/200 ms) (*p* < 0.01 to 0.04, *d* = 0.12 to 0.38) following a tissue flossing intervention (mean pressure 160 ± 3 mmHg) among recreational adult male athletes. The post-intervention RFD changes for tissue flossing were also greater than (i.e., significantly different than) the static stretching (*p* < 0.05) and non-treatment comparison groups (*p* < 0.05). A study by Galis & Cooper [25] examined the immediate post-intervention changes in ankle plantar flexion and dorsiflexion IPT and dorsiflexion power at three different band stretch pressures: control (mean 4.70 ± 1.06 mmHg), FLOSS_150mmHg_ (mean 152.00 ± 4.45 mmHg), and FLOSS_200mmHg_ (203.00 ± 7.35 mmHg) among university adult athletes. For plantar flexion IPT, the researchers documented non-significant (*p* > 0.05) post-intervention changes among the FLOSS_150_ (*d* = 0.02)_,_ FLOSS_200_ (*d* = −0.16), and control groups. For dorsiflexion IPT, the researchers reported non-significant (*p* > 0.05) post-intervention changes among the FLOSS_150_ (*d* = 0.18), FLOSS_200_ (*d* = −0.05), and the control group. For dorsiflexion power, the researchers reported non-significant (*p* > 0.15) but small post-intervention effects among the FLOSS_150_ (*d* = 0.29) when compared to the FLOSS_200_ (*d* = −0.15) and the control group [25]. A study by Kalc et al. [20] reported non-significant post-intervention changes after tissue flossing to the soft tissue for MVIC ankle plantarflexion (*p* = 0.203) and no changes at a 10 min follow-up (*p* = 0.568) among young martial arts athletes (e.g., ~16 years of age). However, the researchers did report a significant reduction in H reflex activity after tissue flossing to the calf. The researchers also found no post-intervention changes in wrapping the ankle joint, as documented in the prior section (Table 3) [20]. 

### 3.4. Jump Performance 

Eight studies investigated the post-intervention changes in a tissue flossing intervention on measures of jump performance for the ankle, thigh, and calf [23,26,27,28,29,30,31,32] (Table 2). 

#### 3.4.1. Tissue Flossing: Joint

Four studies reported using a single-session tissue flossing intervention on the ankle [26,27,30] and knee joint [24]. Three studies included a calculated band stretch pressure of ≥178 mmHg on the ankle joint, which was directly measured and monitored by a sub-band digital pressure sensor (e.g., Kikuhime™ pressure monitor, TT MediTrade, Sorø, Denmark) [26,27,30]. One study only used the recommended stretch length (e.g., 50% band maximal length) and did not document any methods for measuring or monitoring band stretch pressure [24]. Two studies used the double-leg CMJ [27,30], one study used the single-leg CMJ [26], and one study used SLTHT [24] to measure jump performance. The total tissue flossing exercise intervention time ranged from 2 to 3 min [24,26,27,30]. All studies used a comparison group (Table 2).

Regarding results, one study reported significant post-intervention improvements and three studies reported non-significant changes but small effects after a tissue flossing intervention. For the *ankle joint*, Driller & Overmayer [26] reported immediate post-intervention improvements (*p* < 0.01) (e.g., within 5 min) after a tissue flossing intervention (mean pressure 182 ± 38 mmHg) for single-leg CMJ height (*p* < 0.01, *d* = 0.28) and velocity (*p* < 0.01, *d* = 0.22) when compared to the control condition among recreational adult athletes. Driller et al. [27] conducted a follow-up study measuring post-intervention changes in CMJ after a tissue flossing intervention (mean pressure 178 ± 18 mmHg) at four time points: 5 min, 15 min, 30 min, and 45 min. The researchers reported non-significant changes (*p* = 0.21) for all time points compared to the control group among the same athletes from the prior study. However, there was a small effect at 30 min (*d* = 0.32) and 45 min (*d* = 0.21) post-intervention [27]. Mills et al. [30] reported non-significant post-intervention changes (*p* > 0.05) but a small effect (*d* = 0.28) in double-leg CMJ 5 min after a tissue flossing intervention (mean pressure 178 ± 22 mmHg). The researchers compared tissue flossing to the control condition among professional adult male rugby athletes (Table 3). 

For the *knee joint*, Wu et al. [24] reported non-significant post-intervention changes (*p* > 0.05) after a tissue flossing intervention to the knee joint immediately and at the 20-min follow-up with SLTHT performance when compared to the elastic band condition among healthy adult women. 

#### 3.4.2. Tissue Flossing: Soft Tissue

Four studies reported using a single-session tissue flossing intervention on the thigh [23,29,31], or calf [28]. Three studies [23,29,31] used a calculated band stretch pressure ≥ 95 mmHg on the thigh, which was directly measured and monitored by a sub-band digital pressure sensor (e.g., PicoPress™ pressure sensor, Microlab Ellettronica Sas, San Nicolò PD, Italy; Tekscan pressure sensor, Tekscan, South Boston, MA, USA) or an adapted sphygmomanometer. One study [28] only used the recommended stretch length (e.g., 50% band maximal length) and did not document any methods for measuring or monitoring band stretch pressure. 

Three studies [23,28,31] used the double-leg CMJ and one study [29] used the single-leg CMJ to measure jump performance. All studies used a comparison group that included a non-treatment control group [23,29,31], a sham group [29], or a comparison group/condition (e.g., self-myofascial rolling, warm-up session) [28,31]. The total tissue flossing exercise intervention time ranged from 2 to 6 min [23,28,29,31]. All studies used a comparison group (Table 2).

Regarding results, three studies reported non-significant post-intervention changes, and one reported significant change after a tissue flossing intervention. For the *thigh region*, Maust et al. [29] reported immediate non-significant post-intervention changes after a tissue flossing intervention (pressure range of 140 to 200 mmHg) with single-leg CMJ performance (*p* = 0.18) when compared to sham (pressure range 10 to 40 mmHg) and control conditions among recreationally active adults. Konrad et al. [23] documented non-significant immediate post-intervention changes after a tissue flossing intervention (mean pressure 154.3 ± 13.3 mmHg) with double-leg CMJ performance (*p* = 0.75) when compared to the non-treatment control group among adult men. Paravlic et al. [31] investigated the post-intervention changes after a tissue flossing intervention with double-leg CMJ performance after three conditions: warm-up (dynamic), tissue flossing, and a non-treatment control condition among recreational adult university athletes. The researchers used six post-intervention measurement points for CMJ (0.5, 3, 6, 9, 12, and 15 min). The researchers reported that the tissue flossing intervention (mean pressure 95 ± 17.4 mmHg) demonstrated negative post-intervention changes for double-leg CMJ height (*p* < 0.001) and average power (*p* < 0.001) when compared to the control. However, the warm-up (dynamic) condition produced positive post-intervention changes in double-leg CMJ height (*p* < 0.001) and average power (*p* < 0.001) when compared to the control condition. 

For the *calf region*, Klich et al. [28] measured the post-intervention changes for double-leg CMJ height after a tissue flossing and self-myofascial rolling intervention at five-time points (5, 15, 30, 45, and 60 min) among adult male university athletes. The researchers reported significant post-intervention changes after tissue flossing for the 15- to 60-min time points (*p* ≤ 0.001). There were non-significant post-intervention changes for the self-myofascial rolling condition (*p* > 0.05) (Table 3).

### 3.5. Sprint Performance

Three studies investigated the post-intervention effects of a tissue flossing intervention on measures of sprint performance for the ankle and calf [26,27,28] (Table 2).

#### 3.5.1. Tissue Flossing: Joint

Two studies [27,30] reported using a single-session tissue flossing intervention on the ankle joint. Both studies used a calculated band stretch pressure ≥178 mmHg on the ankle joint, which was directly measured and monitored by a sub-band digital pressure sensor (e.g., Kikuhime™ pressure monitor, TT MediTrade, Sorø, Denmark). Both studies measured 5 m to 20 m sprint performance. The total tissue flossing exercise intervention time among studies was 2 min [27,30]. All studies used a comparison group (Table 2). 

Regarding results, both studies reported non-significant post-intervention changes after a tissue flossing intervention but small effects. Driller et al. [27] reported measuring post-intervention changes in sprint performance (5 m to 15 m) after a tissue flossing intervention (mean pressure 178 ± 18 mmHg) at four time points: 5 min, 15 min, 30 min, and 45 min among recreational adult athletes. The researchers found non-significant changes (*p* > 0.05) for all time points when compared to the control group. However, there was a small effect at all time points (*d* = −0.21 to −0.27) post-intervention [27]. Mills et al. [30] reported measuring post-intervention changes in 5 m to 20 m sprint performance (split times) after a tissue flossing intervention (mean pressure 178 ± 22 mmHg) at two time points: 5 min and 30 min on professional adult male rugby athletes. The researchers found non-significant post-intervention changes (*p* > 0.05) but small effects for 10 m (*d* = −0.45) and 15 m (*d* = −0.24) sprint time 30 min post-intervention when compared to the control condition (Table 3). 

#### 3.5.2. Tissue Flossing: Soft Tissue

One study reported using a single-session tissue flossing intervention on the calf region [28]. They did not document any method for measuring or monitoring band stretch pressure [28]. The study used a 15 m sprint test to measure performance. The total tissue flossing exercise intervention time was 2 min [28]. The study used a comparison group (Table 2). 

Regarding results, Klich et al. [28] measured the post-intervention changes after a tissue flossing intervention and self-myofascial rolling on 15 m sprint performance at five time points: 5 min, 15 min, 30 min, 45 min, and 60 min among adult male university athletes. The researchers found significant post-intervention changes and small effects for the tissue flossing intervention at post-intervention 15 m sprint for all time points up to 60 min (*p* ≤ 0.001). Post-intervention changes occurred at all time points from up to 30 min for self-myofascial rolling (*p* ≤ 0.001) (Table 3).

### 3.6. Balance and Agility 

Four studies investigated the post-intervention effects of a tissue flossing intervention on measures of balance and agility for the knee, ankle, and calf [18,32,33,34] (Table 2). 

#### Tissue Flossing: Joint and Soft Tissue

Two research groups applied the tissue flossing band with a hand-held dynamometer to measure stretch force to 40 N but did not report any monitoring of sub-band pressure during the intervention [17,32]. Two studies only used the recommended stretch length (e.g., 50% band maximal length) and did not document any methods for measuring or monitoring band stretch pressure [24,33].

The studies used the single-leg balance test (SLBT) [24,33] for static balance assessment, the Y-balance Test (YBT) [17,33] for dynamic balance assessment, and the figure-of-eight hop test (FHT) for agility [32]. The LESS test was also used to assess jump landing stabilization performance [24]. The total tissue flossing intervention time among studies ranged from 2 to 3 min [17,24,32,33]. All studies used a comparison group (Table 2). 

Regarding results, the studies reported mixed post-intervention changes after a tissue flossing intervention on specific measures of balance and agility. Moon et al. [33] reported immediate post-intervention changes after a tissueflossing intervention to the ankle joint with the SLBT (*p* < 0.05) and the YBT (*p* < 0.05) among healthy adults. The researchers also used a comparison exercise condition that produced post-intervention changes (*p* < 0.05) in static and dynamic balance. Chang et al. [17] reported significant post-intervention changes from a tissue flossing intervention to the knee joint in YBT performance immediately (*p* ≤ 0.001, *d* = 0.57) and at a 20-min follow-up (*p* ≤ 0.001, *d* = 0.86) among active adult men. When compared to an elastic bandage, tissue flossing showed greater post-intervention improvements in YBT performance immediately (*p* = 0.016, *d* = 0.85) and at the 20-min follow-up (*p* = 0.004, *d* = 0.66). Wu et al. [24] reported non-significant post-intervention changes (*p* > 0.05) after a tissue flossing intervention to the knee joint immediately and at the 20-min follow-up with SLBT performance when compared to the elastic band condition among healthy adult women. However, tissue flossing produced a significantly (*p* = 0.032) higher LESS score at the 20-min follow-up when compared to the elastic band condition. 

Huang et al. [32] measured the post-intervention changes after a tissue flossing intervention to the ankle joint and calf region on FHT performance among recreational adult women. The researchers measured three post-intervention time points: 5 min, 30 min, and 60 min. The researchers compared three groups: ankle, calf, and exercise only. The researchers reported significant post-intervention changes for the tissue flossing ankle group at the 5 min (*p* < 0.001, *d* = 0.39), 30 min (*p* = 0.004, *d* = 0.44), and 60 min (*p* = 0.007, *d* = 0.45) follow-up time points. The tissue-flossing calf group only showed improvements at 5 min (*p* = 0.04, *d* = 0.29) and the exercise-only condition produced significant post-intervention changes at 30 min (*p* = 0.02, *d* = 0.35) and 60 min (*p* = 0.04, *d* = 0.42) [32] (Table 3).

## 4. Discussion

This systematic review appraised the research evidence regarding the post-intervention effects of tissue flossing on athletic performance measures that included muscle strength, jump performance, sprint performance, balance, and agility. The type of tissue flossing intervention technique (e.g., joint and soft tissue) was also appraised for each study. Eighteen studies qualified for this analysis, all of which contained different methodologies. The accepted studies were categorized and analyzed by the athletic performance measure and the type of tissue flossing intervention. 

The research regarding tissue flossing on muscle strength measures has the most evidential support (n = 6) followed by the jump performance (n = 2), balance performance (n = 2), agility (n = 2), and sprint performance (n = 1). Overall, there was a mix of studies documenting significant and non-significant post-intervention changes across athletic performance measures. The following sections will further discuss the results as they apply to healthcare professionals.

### 4.1. Muscle Strength 

Nine studies investigated the post-intervention effects of a single-session tissue flossing intervention on various measures of strength [17,18,19,20,21,22,23,24,25]. The research documented mixed results regarding the immediate and up to 45 min post-intervention effects of tissue flossing of the joint and soft tissue. For the *joint*, two studies reported significant post-intervention changes with small to large effects after tissue flossing to the knee [17] and shoulder [16]. For *soft tissue*, four studies documented significant post-intervention changes with small to large effects after tissue flossing to the thigh [18,22,23] and calf [21]. Two studies reported non-significant post-intervention changes with tissue flossing to the ankle joint [19,20] and calf [20]. One study [25] reported non-significant post-intervention changes but small effects, which they considered an improvement for one strength measure (e.g., ankle dorsiflexion power). 

Tissue flossing band stretch pressure is a treatment parameter that sports medicine professionals should consider as to how it would affect muscle strength. Six studies [18,20,21,22,23,25] reported using a digital sub-band pressure sensor or adapted sphygmomanometer to measure and monitor band stretch pressure during the intervention. Three studies [16,17,35] did not measure or monitor band stretch pressure. Interestingly, a larger number of studies reporting significant post-intervention changes came from researchers who measured and monitored band stretch pressure (n = 4) (mean pressure 100 to 154.3 mmHg) versus studies that did not monitor pressure (n = 2). Among those studies that determined and monitored pressure, it appears that non-significant post-intervention effects occur with higher band stretch pressure (≥200 mmHg), which may create a neuromuscular inhibitory effect [20,25]. Kalc et al. [20] also reported reduced soleus H reflex activity after a tissue flossing intervention to both the ankle and calf with a higher band stretch pressure (≥230 mmHg). All studies included an exercise intervention such as active joint motion, active closed chained movements (e.g., squats, lunges), or no motion. The total tissue flossing intervention time among studies ranged from 2 min to 10 min, which included sets and repetitions for the different movements. 

In summary, healthcare professionals should consider the emerging research evidence supporting tissue flossing for enhancing short-term muscle strength. The majority of appraised studies (n = 6) support the efficacy of tissue flossing on the joint or soft tissue. A single session of tissue flossing may produce small to large post-intervention effects (*d* > 0.2) in measures of muscle strength of the shoulder, knee, thigh, and calf. Tissue flossing is typically combined with an exercise intervention of ≥2 min. The post-intervention changes in muscle strength may last up to 45 min, but the effects may begin to diminish after 20 min and beyond [16,17]. Tissue flossing band stretch pressure may influence the effects of the intervention and should be determined and monitored during treatment [8,25]. Professionals should consider that higher band pressures (e.g., >200 mmHg) may produce negative effects versus lower pressure, which may produce more favorable post-intervention effects. This needs to be considered when using tissue flossing for athletic performance.

### 4.2. Jump Performance

Seven studies investigated the post-intervention effects of a tissue flossing intervention on the joint or soft tissue on measures of jump performance [23,26,27,28,29,30,31]. The research documented mixed results regarding the immediate and up to 60 min post-intervention effects of tissue flossing to the joint or soft tissue.

For the *joint*, one study [26] reported significant post-intervention changes with small effects after tissue flossing to the ankle on jump performance. Two studies [27,30] reported non-significant post-intervention changes but small effects on jump performance, which the researchers considered as an improvement. For *the soft tissue*, one study [28] reported significant post-intervention changes after tissue flossing to the calf on jump performance. Three studies [23,29,31] reported non-significant post-intervention changes after a tissue flossing intervention to the thigh. Tissue flossing band stretch pressure should also be considered since it may affect jump performance. Five studies [26,27,29,30,31] reported using a digital sub-band pressure sensor to measure and monitor band stretch pressure. One study did not determine or monitor band stretch pressure [28]. Among studies measuring and monitoring band stretch pressure, only one study [26] reported significant post-intervention changes (mean pressure 182 mmHg) with tissue flossing to the ankle. The other four studies reported non-significant changes and did monitor band stretch pressure (mean range of 95 to 200 mmHg). Interestingly, the study that did not measure or monitor band pressure reported significant post-intervention changes after tissue flossing to the calf [28]. All studies included an exercise intervention such as active joint motion and active closed chained movements (e.g., squats, lunges). The total tissue flossing intervention time among studies was 2 min, which included sets and repetitions for the different movements. 

In summary, healthcare professionals should consider the mixed tissue flossing research evidence on jump performance. Most studies (n = 4) reported non-significant post-intervention effects after tissue flossing to the joint and soft tissue. The two supporting studies suggest that a single session of tissue flossing (2 min) to the ankle joint or calf may produce small immediate post-intervention effects in jump performance [26,28]. One study monitored band pressure [26] and one did not monitor pressure [28]. Healthcare professionals should consider that jump testing requires complex movement involving different upper and lower extremity joints, muscle groups, and muscles of the core. Tissue flossing to a single joint or soft-tissue region of the lower body may not have created a strong enough stimulus to induce changes during a complex movement [23]. This idea may be supported by the majority of studies reporting non-significant post-intervention changes. Healthcare professionals should consider the implications of wrapping a single joint or soft-tissue region versus multiple areas before complex movements (e.g., jumping) since a greater stimulus may be needed to maximize the effects of the intervention. Wrapping stretch pressure should also be considered since it may influence post-intervention outcomes. Further research is necessary to confirm these findings due to the mixed results among published studies. 

### 4.3. Sprint Performance 

Three studies investigated the post-intervention effects of a tissue flossing intervention on the ankle joint or calf on measures of sprint performance (5, 10, 15, 20 m) [26,27,28]. The researchers documented mixed results up to 60 min post-intervention. For *the joint*, two studies [27,30] reported non-significant post-intervention changes but small effects after tissue flossing to the ankle joint. Both studies determined and monitored band stretch pressure (mean pressure 178 mmHg). For *soft tissue*, one study [28] reported significant post-intervention changes and small effects after tissue flossing. However, they did not measure or monitor sub-band stretch pressure. All studies included an exercise intervention such as active joint motions and active closed chain movements. The total tissue flossing intervention time among studies was 2 min, which included sets and repetitions for the different movements.

In summary, the research evidence on the effects of tissue flossing on sprint performance is mixed, with few published studies. Only one study reported significant post-intervention changes after tissue flossing to the calf. Klich et al. [28] suggest that a single session of tissue flossing (2 min) may produce significant post-intervention changes and small effects in sprint performance from 15 min to 60 min after treatment. However, they did not measure or monitor band stretch pressure. Healthcare professionals should also consider that sprint testing requires complex movement involving different upper and lower extremity joints, muscle groups, and muscles of the core. Tissue flossing to a single joint or soft-tissue region of the lower body may not have created a strong enough stimulus to induce post-intervention changes during testing [23]. Healthcare professionals should consider the implications of wrapping one body region before conducting complex athletic performance tests such as jumping and sprinting. It is important to consider that only three studies have been published (to date) on sprinting and more investigations are needed to further validate these findings. 

### 4.4. Balance and Agility 

Three studies [17,24,33] investigated the post-intervention effects of a tissue flossing intervention on the joint on measures of static and dynamic balance. Two studies [17,33] reported significant post-intervention changes with moderate to large effects after a tissue flossing intervention to the knee and ankle for single-leg balance and the Y-balance Test. One study used a hand-held dynamometer to measure band application stretch resistance (40 N) [17] and one study used the recommended band stretch length [33]. No studies monitored band pressure during treatment. One study [24] reported non-significant changes after tissue flossing to the ankle joint, except at a 20 min follow-up for the LESS test. No published studies on tissue flossing to soft-tissue and measures of balance have been published. All studies included an exercise intervention such as active joint motions and active closed chain movements for a total intervention time of 3 min, which included sets and repetitions for the different movements.

For agility, one study [32] reported significant post-intervention changes with small to medium effects in the figure-of-eight hop agility test up to 60 min after tissue flossing to the ankle joint but only post 5 min after tissue flossing to the calf. Researchers used a hand-held dynamometer to measure band application stretch resistance (40 N) and did not monitor band pressure during treatment. The study included an exercise intervention such as closed chain movements for a total intervention time of 3 sets of 15 reps with 2 min rest between sets.

In summary, the research evidence regarding the effects of tissue flossing on balance and agility is sparse with mixed results. For *balance*, two studies reported significant post-intervention changes and one study documented non-significant changes. All studies wrapped the ankle joint and did not monitor band pressure during the intervention. For *agility*, one study reported significant post-intervention changes after tissue flossing to the ankle joint and calf.

When considering the tissue flossing technique, two research groups [17,32] applied the tissue flossing band to the ankle joint and calf with a hand-held dynamometer to measure stretch force to 40 N and two groups [24,33] only used the recommended stretch length. No studies reported measuring or monitoring sub-band pressure during treatment. Like jump and sprint testing, balance and agility testing often include multiple joints and muscle groups. Tissue flossing to only one joint or soft-tissue region may not influence the outcomes since the technique may not create a strong enough stimulus for observable change. The available research suggests that a single-session tissue flossing intervention to the joint or soft tissue may produce significant post-intervention changes in some measures of balance and agility. However, there are few published studies. Future research is warranted to further validate these findings.

### 4.5. Limitations 

It should be acknowledged that tissue flossing is still an emerging area of research, and this review is limited by our specific search criteria. There are several limitations that warrant discussion. First, all appraised studies used small sample sizes of healthy teen and adult participants, which makes it challenging to generalize the findings beyond the populations studied. Second, the appraised studies included healthy participants with different sport and/or activity levels, such as healthy individuals, recreational athletes, collegiate athletes, and professional athletes. The diversity among participant sport and/or activity levels makes it difficult to directly compare study findings or generalize beyond the populations studied. Third, all studies enrolled either men, women, or both as participants. No studies compared the results of a tissue flossing intervention among the different sexes, which leaves a gap in understanding any unique participant responses. Future direct comparison studies are needed. Fourth, participant demographics (e.g., body fat percentage) were not controlled among the studies in this analysis. Study participants with different body compositions may have experienced diverse responses to the tissue flossing intervention. Fifth, the appraised studies all used different methodologies, making it difficult to directly compare outcomes among studies. The narrative results and discussion attempted to provide pertinent details for healthcare professionals and researchers to consider with each study. Sixth, the systematic search criteria considered tissue flossing studies written in the English language due to limited translational resources and potential difficulty in quality appraisal due to language barriers. This is a limitation since tissue flossing studies written in other languages may exist.

## 5. Clinical Implications

Healthcare professionals should consider that the tissue flossing evidence related to athletic performance measures is still emerging. The current body of evidence is mixed with a diversity of study methodologies (e.g., sample populations, interventions, outcomes), which have produced mixed results. Based upon this analysis, tissue flossing and muscle strength had the best evidence, followed by jump and balance performance. 

When translating the results to practice, healthcare professionals can consider two findings among appraised studies. First, a single tissue flossing treatment ranging from 2 to 10 min that includes an active single joint or active close chain exercise may enhance muscle performance, jump performance, and balance up to 45–60 min post-intervention. Second, tissue flossing interventions on a joint or soft tissue both produced mixed results among studies. A definitive answer on which technique is superior cannot be determined at this time. Future tissue flossing investigations are needed to further investigate these findings.

This section will further discuss clinical implications regarding the tissue flossing technique, flossing band architecture, flossing band stretch pressure, treatment theories, treatment integration, interchangeability, and research reporting.

### 5.1. Tissue Flossing Techniques

The tissue flossing technique is a treatment parameter that healthcare professionals need to consider. The research on both the joint and soft-tissue techniques has produced mixed results with athletic performance measures. Muscle strength has the most evidential support. For muscle strength, six studies reported significant post-intervention effects on performance measures after a tissue flossing intervention to the soft tissue [18,21,22,23] or joint [16,17]. All studies reported measuring initial band stretch pressure and five studies [16,18,21,22,23] monitored band stretch pressure during treatment, which may have influenced the outcomes. Perhaps these significant post-intervention changes are a result of a single body region being treated and tested. Other performance measures such as jumping, sprinting, balance, and agility often include multiple joints and muscles. This may be why the research on these multi-joint, multi-muscle athletic measures is mixed. Tissue flossing to one joint or soft-tissue region may not have a strong enough stimulus to affect surrounding body regions.

To date, a definitive answer on which technique (e.g., joint versus soft tissue) is superior cannot be determined due to the mixed methods and results among published studies and the few direct comparison studies [20,32]. Future comparison research is needed on different subject populations to determine which tissue flossing technique is best. Future research should also attempt to match the optimal tissue flossing technique to the athletic performance measure. Athletic performance measures that involve multiple joints and muscles may need a tissue flossing intervention that covers all involved joints and soft-tissue regions to produce a strong enough stimulus for post-intervention change. For example, when investigating a lower extremity tissue flossing soft-tissue or joint technique on CMJ, the intervention should include wrapping the major soft-tissue (e.g., thigh and calf) or joint (e.g., hip, knee, and ankle) regions of the lower extremity.

### 5.2. Flossing Band Architecture

Many of the tissue flossing studies have used different manufactured tissue flossing bands. Healthcare professionals should consider that the different band architectures (e.g., dimension, strength) can influence the amount of compression the band provides to the myofascia. Among all appraised studies, researchers used tissue flossing bands from three manufacturers that had different strengths from light to heavy (Table 3). Despite researchers measuring and monitoring band pressure, the specific type of band may have effects on study subjects. Thus, the type of tissue flossing bands used in studies may be a confounding variable that researchers should consider. This may be why there are mixed outcomes in the body of tissue flossing research. 

### 5.3. Flossing Band Stretch Pressure 

Healthcare professionals should consider (along with band type) the influence of measuring and monitoring band stretch pressure during treatment to maximize outcomes and ensure patient safety. The majority of muscle strength studies documenting significant post-intervention changes measured and/or monitored band stretch pressure that was ≤180 mmHg. Studies reporting non-significant post-intervention effects in athletic performance measures used higher band stretch pressure (e.g., ≥200 mmHg), which may create a more neuromuscular inhibitory effect [18,20,25]. Kalc et al. [20] also found reduced soleus H reflex activity after a tissue flossing intervention to both the ankle joint and calf with a higher band stretch pressure (≥200 mmHg). Regarding patient safety, researchers have documented that higher band stretch pressure has been linked to different adverse effects such as pain, numbness, loss of ROM, and reduced strength [8,25].

### 5.4. Treatment Theories 

Researchers have postulated that the partial vascular occlusion caused by the tissue flossing band may stimulate different physiological responses upon removal of the band [27]. Physiological responses may include, but are not limited to, reperfusion of blood, altered hormonal responses (e.g., growth hormone, norepinephrine), reflex facilitation, and overall neuromuscular activity [8]. Some researchers have further postulated that ischemic preconditioning results in improved athletic performance [32]. Thus, tissue previously submitted to ischemic conditions will become more resistant to ischemia and its negative effects [36]. The healthcare professional should consider that the amount of tissue flossing band pressure may produce different degrees of vascular occlusion and stimulate specific physiological responses, influencing athletic performance. Perhaps the type of tissue flossing band, treated body part (e.g., joint vs. soft tissue), treatment technique, and band stretch pressure are important factors in creating a strong enough stimulus for observable changes. These theories are still under investigation and future research is needed to validate these concepts. 

### 5.5. Treatment Integration 

Healthcare professionals should consider how they integrate tissue flossing within their comprehensive clinical treatments. Several of the appraised studies in this review suggest that tissue flossing may enhance a warm-up [17,21], an athletic performance, or an injury prevention program [16,22,25,26,28,32,33]. Other appraised studies recommend not including tissue flossing as part of a warm-up due to the potential inhibitory effects it has on muscle activity and overall performance [29,31,35]. Healthcare professionals should use tissue flossing with purpose and sequence it appropriately for their patients’ treatments. Due to this mixed evidence, it must be considered that each patient may respond differently to a tissue flossing intervention. As noted in the prior treatment theory section, variables such as the type of tissue flossing band, treated body part (e.g., joint vs. soft-tissue), treatment technique, and band stretch pressure may influence the treatment stimulus and outcomes. Healthcare professionals should consider the potential benefits and limitations of this intervention. 

### 5.6. Interchangeability 

Another consideration for healthcare professionals and researchers is the possible interchangeability of tissue flossing with other interventions. Several studies from this analysis documented the potential interchangeability between tissue flossing and IASTM, KT, self-myofascial rolling, and different types of stretching (e.g., dynamic) [16,21,22,28]. These data should be considered when performing combined interventions on patients or during a multi-modal treatment program. 

### 5.7. Research Reporting

An important part of improving the body of tissue flossing research evidence is correctly reporting research findings. The reporting and interpretation of tissue flossing results among researchers must be clear. Some researchers [25,27,30] documented non-significant pre- to post-intervention changes with small effects but discussed these results as positive post-intervention changes. The importance and interdependence between statistical significance and effect size should be considered when reporting post-intervention tissue flossing results [34]. Both metrics are complementary and essential in helping the healthcare professional to understand the complete impact of the study findings [37]. Other factors to consider are the study’s descriptive statistics and measurement error, which help the professional interpret and determine the accuracy of the study results.

## 6. Conclusions

Healthcare professionals and researchers should consider the mixed evidence regarding the efficacy of tissue flossing on athletic performance measures. The research on muscle strength measures has the strongest supporting evidence among the appraised studies. The other athletic performance measures, such as jump, sprint, balance, and agility all have mixed outcomes among studies. Perhaps the methodological differences among studies, such as the sample population, types of tissue flossing band, treated body parts (e.g., joint vs. soft tissue), treatment technique, band stretch pressure, and outcome measure, contributed to the mixed evidence. Future controlled, purposeful tissue flossing research is needed in order to address these methodological gaps in the existing body of evidence. Overall, healthcare professionals should consider factors such as the type of flossing band, treated body part, treatment technique, and band stretch pressure when integrating tissue flossing into their patient treatments. 

## Figures and Tables

**Table 1:** Quality assessment of accepted studies.

Author	Study Design	Scale Used	Scale Score	Level of Evidence
Angelopoulos et al. [16]	Randomized controlled trial	DB Checklist	25	Level II
Chang et al. [17]	Randomized crossover design	DB Checklist	25	Level III
Vogrin et al. [18]	Crossover repeated measure design	DB Checklist	23	Level III
Hamadus et al. [19]	Randomized controlled trial design	DB Checklist	23	Level II
Kalc et al. [20]	Randomized crossover repeated measure design	DB Checklist	21	Level III
Kaneda et al. [21]	Non-randomized crossover design	DB Checklist	21	Level III
Kaneda et al. [22]	Randomized crossover design	DB Checklist	22	Level II
Konrad et al. [23]	Randomized crossover design	DB Checklist	20	Level II
Wu et al. [24]	Randomized crossover design	DB Checklist	24	Level III
Galis & Cooper [25]	Randomized controlled trial	DB Checklist	25	Level III
Driller & Overmayer [26]	Randomized crossover design	DB Checklist	21	Level III
Driller et al. [27]	Randomized parallel group design	DB Checklist	21	Level III
Klich et al. [28]	Randomized controlled, repeated measure design	DB Checklist	26	Level II
Maust et al. [29]	Counter-balanced, crossover design	DB Checklist	23	Level III
Mills et al. [30]	Counter-balanced, crossover design with experimental and control trials	DB Checklist	23	Level III
Paravlic et al. [31]	Randomized, crossover, counterbalanced repeated measures design	DB Checklist	25	Level II
Huang et al. [32]	Randomized crossover design	DB Checklist	24	Level II
Moon et al. [33]	Randomized crossover design	DB Checklist	24	Level II

DB Checklist: Downs and Black Checklist; level of evidence: OCEMB criteria.

**Table 2:** Characteristics of accepted studies.

Authors	Participants	Body Region	Band Type and Technique	Intervention	Comparison	Outcomes	Key Findings
Angelopoulos et al. [16]	80 healthy amateur overhead adult athletes (age 23.03 ± 1.89) TF: 20; IASTM: 20 IASTM + TF: 20 KT: 20	Shoulder joint (dominant)	Brand: Kinetic Floss (strength and size not reported) Technique: not described (image shows 50% overlap pattern).	TF to shoulder with PROM, AROM, and 1 kg ball toss for 10 min.	IASTM KT IASTM + TF Control (non-dominant) 10 min treatment each.	IPT and total work. One-arm seated shot put test.	All groups showed equally significant changes immediately and post 45 min for the IPT, TW, and OASPT scores when compared to non-dominant control.
Chang et al. [17]	30 healthy recreational active adult men (age 24.1 ± 1.7)	Knee joint	Brand: Sanctband COMPRE Flossband Green (light—5 cm) Technique: 50% overlap pattern.	TF to knee with CKC for 3 min.	EB with CKC for 3 min.	IPT Knee Proprioception YBT	TF showed greater significant changes immediately and post 20 min in IPT and YBT scores when compared to EB. No changes in proprioception.
Wu et al. [24]	20 healthy adult university women (age 21.8 ± 2.31)	Knee joint	Brand: Sanctband COMPRE Flossband Green (light—5 cm) Technique: 50% overlap pattern.	TF to knee with CKC for 3 min. * All subjects: pre-test warm-up for 5 min.	EB to knee with CKC for 3 min.	SLBT SLTHT LESS Test	Both TF and EB showed equal non-significant changes immediate and post 20 mins for SLBT and SLTHT. TF showed significantly greater LESS scores at post 20 min than EB.
Kalc et al. [20]	11 healthy young elite martial arts athletes (5 men/6 women) (age 16.6 ± 1.2)	Ankle joint Calf region	Brand: Medical Flossing Band (5.5 cm, strength not reported) Technique (ankle): Figure 8 pattern with 50% overlap Technique (calf): 50% overlap pattern.	TF to ankle. TF to calf. Both groups did no movement for 6 min (3 sets of 2 min).	Control: intervention only for 6 min.	MVIC H reflex	All groups produce non- significant changes immediately and post 10 min in MVIC. H reflex decreased with TF to ankle and calf up to 10 min post. TF to calf resulted in a higher H reflex reduction.
Driller & Overmayer [26]	52 healthy recreational adult athletes (29 men/23 women) (age 20.0 ± 4.0) TF: 26; Control: 26	Ankle joint	Brand: Life Flossband (strength and size not reported) Technique: Figure 8 pattern with 50% overlap.	TF to ankle with AROM for 2 min.	Control: AROM only for 2 min.	Single-leg CMJ	TF showed significant immediate changes in single-leg CMJ height and velocity. No significant changes in the control.
Driller et al. [27]	69 healthy recreational adult athletes (32 men/37 women) (age 19.0 ± 2.0) TF: 38; Control: 31	Ankle joint	Brand: Life Flossband (band strength and size not reported) Technique: Figure 8 pattern with 50% overlap.	TF to ankle with AROM for 2 min. * All subjects: pre-test warm-up for 5 min.	Control: AROM only for 2 min.	CMJ 15 m sprint test	The TF group showed small but non-significant changes at all time points up to 45 min in CMJ and sprint times when compared to control. A small effect was found for both outcomes at 30 min and 45 min post.
Mills et al. [30]	14 healthy professional adult male rugby athletes (age 23.9 ± 2.7)	Ankle joint	Brand: Life Flossband (strength and size not reported) Technique: Figure 8 pattern with 50% overlap.	TF to ankle with AROM for 2 min. * All subjects: pre-test warm-up for 5 min.	Control: AROM only for 2 min.	CMJ 5–20 m sprint test	TF showed no significant changes post 5 min and post 30 min for CMJ and sprint times when compared to the control. TF showed small effect sizes for CMJ at post 5 min and for 10 m and 15 m sprint times at post 30 min.
Huang et al. [32]	18 healthy recreational adult women athletes (age: 22.39 ± 2.73)	Ankle joint Calf region	Brand: Sanctband COMPRE Flossband Plum (Heavy—size not reported) Technique (ankle): Figure 8 pattern with 50% overlap. Technique (calf): 50% overlapping pattern.	TF to calf and ankle with CKC. * All subjects: pre-test warm-up for 2 × 10 reps.	CKC exercise only (3 × 15 reps).	FHT	TF to ankle showed significant changes in FHT agility up to post 60 min. TF to calf showed significant changes only at post 5 min. CKC exercise only resulted in significant changes at post 30 and post 60 min.
Moon et al. [33]	25 healthy adults (age 29.4 ± 3.6)	Ankle joint	Brand: Sanctband COMPRE Flossband Green (light—5 cm) Technique: Figure 8 pattern with 50% overlap.	TF to ankle with AROM for 2 min.	Control: AROM only for 2 min.	SLBT YBT	Both TF and control showed significant changes at post 20 min and post 40 min in SLBT and YBT scores.
Paravlic et al. [31]	19 healthy recreational adult university athletes (11 males, 8 females) (age 23.1 ± 2.7)	Thigh region	Brand: Sanctband COMPRE Flossband Plum (heavy—5 cm) Technique: 50% overlap pattern.	TF to thigh with AROM and CKC for 6 min (3 × 2 min).	Control: TF to thigh with low band pressure with AROM and CKC for 6 min. Warm-up: bike, dynamic stretching, and CMJ for 5 min.	CMJ	TF flossing resulted in non-significant changes in CMJ at all post time points when compared to control. The warm-up condition results in significant changes in CMJ at all post time points.
Maust et al. [29]	21 healthy recreationally active adults (8 men/13 women) (age 22.62 ± 2.99)	Thigh region	Brand: not reported (10.16 cm—strength not reported) Technique: 50% overlap pattern.	TF to thigh with AROM and CKC for 6 min (3 × 2 min). * All subjects: pre-test warm-up for 5 min.	TF sham with AROM and CKC for 6 min. Control: AROM and CKC only for 6 min.	Single-leg CMJ	TF showed non-significant immediate changes in single-leg CMJ when compared to control and sham conditions.
Hamadus et al. [19]	66 healthy recreational adult athletes (21 men/45 women) (age 21.4 ± 2.15) Warm-up + TF: 36 Control: 30	Thigh region	Brand: Sanctband COMPRE Flossband Plum (heavy—5 cm) Technique: 50% overlap pattern.	Warm-up exercises with TF to thigh. * All subjects: pre-test warm-up for 8 min.	Control: warm-up only with no TF.	IPTP, total work, and power	Both TF and control showed non-significant immediate changes in IPTP, total work, and power.
Kaneda et al. [21]	20 healthy recreational adult male athletes (age 22.5 ± 1.0)	Calf region	Brand: Sanctband COMPRE Flossband Blueberry (medium—5 cm) Technique: 50% overlap pattern.	TF to calf with AROM and PROM for 4 min (2 × 2 min, 2 min rest in between).	Static stretching for 5 min. Control: rest 6 min.	MVIC RFD	TF showed greater significant changes at post 5 min in MVIC and RFD when compared to static stretching.
Kaneda et al. [22]	17 healthy adult men (age 23.2 ± 1.1)	Thigh region	Brand: Sanctband COMPRE Flossband Blueberry (medium—5 cm) Technique: 50% overlap pattern.	TF with AROM and PROM for 4 min.	Dynamic stretching for 4 min. Control: AROM 20 × 2 sets (2 min rest between sets).	MVIC MVEC RFD	TF showed greater significant immediate post changes in MVEC than the dynamic stretching and control condition.
Konrad et al. [23]	16 healthy adult men (age 25.69 ± 4.1)	Thigh region	Brand: Artzt Vitality Flossband (standard—5.5 cm) Technique: 50% overlap pattern.	TF to thigh with CKC for 2 min.	Control: CKC for 2 min without TF.	MVIC CMJ	TF showed significant immediate post changes in MVIC but non-significant post changes for CMJ when compared to control.
Vogrin et al. [18]	19 healthy recreationally trained adults (14 men/5 women) (age 23.8 ± 4.8)	Thigh region	Brand: Medical Flossing Band (5 cm-strength no reported) Technique: 50% overlap pattern.	TF thigh low: (100–140 mmHg). TF thigh high: (150–210 mmHg). * groups used AROM for 6 min. * All subjects: pre-test warm-up for 5 min.	Control: TF (20 mmHg) to thigh with AROM for 6 min.	MVIC	TF low pressure showed significant changes immediately and post 30 min in MVIC of knee extensors when compared to the TF high-pressure condition and control.
Galis & Cooper [25]	30 healthy university adults (16 men/14 women) (age 21.50 ± 2.57)	Calf region	Brand: Medical Flossing Band (5 cm-strength no reported) Technique: 50% overlap pattern.	TF calf (150 mmHg). TF calf (200 mmHg). * both groups used AROM and CKC for 2 min. * All subjects: pre-test warm-up for 5 min.	Control: TF to calf (<5 mmHg) with AROM and CKC for 2 min.	IPTP	TF (150), TF (200), and control showed non-significant immediate changes for IPTP plantar flexion. TF (150) showed non-significant immediate changes for IPTP dorsiflexion but a small effect when compared to the TF (200) and control.
Klich et al. [28]	32 healthy adult male university athletes (age 22 ± 1.5)	Calf region	Brand: Artzt Vitality Flossband (strength and size not reported) Technique: 50% overlap pattern.	TF to calf with AROM and CKC for 2 min. * All subjects: pre-test warm-up for 5 min.	Self-myofascial rolling (3 sets of 30 ss) Control: rest	CMJ 15 M sprint	TF showed significant changes in CMJ from post 15 min to post 60 min. TF showed significant changes in sprint times for all time points up to post 60 min. Self-myofascial rolling showed significant changes in sprint times at post 5 min to post 30 min.

TF: tissue flossing; AROM: joint active range of motion; CMJ: counter movement vertical jump; CKC: closed kinetic chain movements; EB: elastic bandage; FHT: figure-of-eight hop test; IPTP: isokinetic peak torque and/or power; IASTM: instrument-assisted soft-tissue mobilization; KT: kinesiology tape; MVIC: maximum voluntary isometric contraction; MVEC: maximum voluntary eccentric contraction; OASPT: one-arm shot put test; PROM: joint passive range of motion; RFD: rate of force development; SLBT: single-leg balance test; SLTHT: single-leg triple-hop test; TW: total work; YBT: Y-balance Test; * Pre-test warm-up (all subjects): one or more of the following: running, bike, dynamic movements, CMJ, trampoline jumps, and CKC.

**Table 3:** Study intervention and outcome measures.

Authors	Participants	Body Region	Total Intervention Time	Stretch Pressure or Tension (mmHg or N)	Single Session	Sub-Band Pressure Sensor	Outcomes Measures				
							Muscle	Jumping	Sprint	Balance	Agility
Angelopoulos et al. [16]	Amateur adult overhead athletes	Shoulder joint	Total: 10 min 2 min each of AROM, PROM, and ball toss. 2 min rest between exercises.	160–180 mmHg	Yes	Yes	↑				
Chang et al. [17]	Recreationally active adult men	Knee joint	Total: 3 min 10 reps each @ 30 BPM of CKC	* 40 N (stretch tension with HHD)	Yes	No	↑			↑	
Wu et al. [24]	Women adult university students	Knee joint	Total: 3 min 10 reps each @ 30 BPM of CKC	50% band stretch length	Yes	No		↓		↓	↑
Kalc et al. [20]	Young elite martial arts athletes	Ankle joint Calf region	Total: 6 min 3 × 2 min no exercise	* 293.93 ± 54.7 mmHg (ankle) * 233.6 ± 52.6 mmHg (calf)	Yes	Yes	↓				
Driller & Overmayer [26]	Recreational adult athletes	Ankle joint	Total: 2 min 20 reps of AROM	* 182 ± 38 mmHg	Yes	Yes		↑			
Driller et al. [27]	Recreational adult athletes	Ankle joint	Total: 2 min 20 reps of AROM	* 178 ± 18 mmHg	Yes	Yes		↓	↓		
Mills et al. [30]	Male professional adult rugby athletes	Ankle joint	Total: 2 min Joint AROM	~180 mmHg	Yes	Yes		↓	↓		
Huang et al. [32]	Recreational adult women athletes	Ankle joint Calf region	Total: 3 × 15 reps KC with 2 min rest between sets	40 N (stretch tension with HHD)	Yes	No					↑
Moon et al. [33]	Healthy adults	Ankle joint	Total: 2 min Joint AROM	50% band stretch length	Yes	No				↑	
Paravlic et al. [31]	Recreational adult university athletes	Thigh region	Total: 6 min 3 × 2 min 10 reps each of CKC and 15 reps of joint AROM	Flossing: 95 ± 17.4 mmHg Control: 18.9 ± 3.5 mmHg	Yes	Yes		↓			
Maust et al. [29]	Recreational active adults	Thigh region	Total: 2 min 20 reps each of CKC movement and joint AROM	Flossing: 140–200 mmHg Sham: 10–40 mmHg	Yes	Yes		↓			
Hamadus et al. [19]	Recreational adult athletes	Thigh region	Total: 8 min Warm-up exercises	50% band stretch length	Yes	No	↓				
Kaneda et al. [22]	Recreational male athletes	Thigh region	Total: 4 min (2 × 2 min) 20 reps each of AROM and 4 reps of PROM. 2 min rest between sessions.	* 134.1 ± 10.2 mmHg	Yes	Yes	↑				
Kaneda et al. [21]	Healthy adult men	Calf region	Total: 4 min (2 min × 2) 20 reps each of AROM and 4 reps of PROM. 2 min rest between sessions.	* 160 ± 3 mmHg	Yes	Yes	↑				
Konrad et al. [23]	Healthy adult men	Thigh region	Total: 2 min 20 reps CKC movements	* 154.3 ± 13.3 mmHg	Yes	Yes	↑	↓			
Vogrin et al. [18]	Recreational trained adults	Thigh region	Total: 6 min 3 sets of 2 min AROM. 90 s rest in between sessions.	High: 150–210 mmHg Low: 100–140 mmHg Control: 20 mm Hg	Yes	Yes	↑				
Galis & Cooper [25]	Healthy university adults	Calf region	Total: 2 min AROM + 20 reps CKC	Group 1: 150 mmHg Group 2: 200 mmHg Control: <5 mmHg	Yes	Yes	↓				
Klich et al. [28]	Healthy male adult university athletes	Calf region	Total: 2 min 20 reps CKC	50% band stretch length	Yes	No		↑	↑		

* Mean values; AROM: joint active range of motion; CKC: closed kinetic chain movements; HHD: hand-held dynamometer; PROM: joint passive range of motion of joint.; ↑ = significant post-intervention changes; ↓ = non-significant post-intervention changes.

## Data Availability

Data analyzed for this systematic review are from the published manuscripts appraised in the manuscript. These studies and data are available on electronic databases or the respective journal websites.
